# Flexibility and Strength Effects of Adapted Nordic Walking and Myofascial Exercises Practice in Breast Cancer Survivors and Analysis of Differences

**DOI:** 10.3390/healthcare12020222

**Published:** 2024-01-16

**Authors:** Teresa Morano, Federica Lancia, Alessandra Di Marco, Gianluca Viscioni, Ines Bucci, Simona Grossi, Raffaello Pellegrino, Lucia Cugusi, Antonino Grassadonia, Andrea Manca, Valentina Bullo, Riccardo Di Giminiani, Pascal Izzicupo, Angela Di Baldassarre, Andrea Fusco, Cristina Cortis, Giorgio Napolitano, Andrea Di Blasio

**Affiliations:** 1 Department of Medicine and Aging Sciences, “G. d’Annunzio” University of Chieti-Pescara, Via Polacchi L. 11, 66100 Chieti, Italy; moranoteresa@gmail.com (T.M.); lanciafederica@gmail.com (F.L.); diemmealessandra@gmail.com (A.D.M.); ines.bucci@unich.it (I.B.); pascal.izzicupo@unich.it (P.I.); angela.dibaldassarre@unich.it (A.D.B.); giorgio.napolitano@unich.it (G.N.); 2 Department of Neuroscience, Biomedicine and Movement Sciences, University of Verona, Piazzale Scuro L.A. 10, 37124 Verona, Italy; gianluca.viscioni@univr.it; 3 Eusoma Breast Center, “G. Bernabeo” Hospital, ASL02 Lanciano-Vasto-Chieti, c.da S. Liberata, 66026 Ortona, Italy; simona.grossi@asl2abruzzo.it; 4 Department of Scientific Research, Campus Ludes, Off-Campus Semmelweis University, 6912 Lugano, Switzerland; raffaello.pellegrino@uniludes.ch; 5 Department of Biomedical Sciences, University of Sassari, Viale San Pietro 43/B, 07100 Sassari, Italy; lucia.cugusi@uniss.it (L.C.); andmanca@uniss.it (A.M.); 6 Department of Innovative Technologies in Medicine and Dentistry, “G. d’Annunzio” University of Chieti-Pescara, Via dei Vestini 31, 66100 Chieti, Italy; antonino.grassadonia@unich.it; 7 Department of Medicine, University of Padova, Via Giustiniani 2, 35128 Padova, Italy; valentina.bullo@unipd.it; 8 Department of Biotechnological and Applied Clinical Sciences, University of L’Aquila, Via Vetoio, 67100 L’Aquila, Italy; riccardo.digiminiani@univaq.it; 9 Department of Human Sciences, Society and Health, University of Cassino and Lazio Meridionale, Via S. Angelo, 03043 Cassino, Italy; andrea.fusco@unicas.it (A.F.); c.cortis@unicas.it (C.C.)

**Keywords:** handgrip test, sit and reach test, workouts

## Abstract

Breast cancer treatments can elicit negative kinesiological side effects concerning both the posture and functional status of breast cancer survivors. As our body is functionally organized in myofascial meridians, physical exercise practice should favor a whole-body approach rather than a local one. The aim of the study was to investigate and compare the effects of two whole-body disciplines, i.e., adapted Nordic Walking and myofascial exercise, on the flexibility and strength performances in BCS. One hundred and sixty breast cancer survivors were trained three times per week for 12 weeks through adapted Nordic Walking or myofascial exercise. Handgrip, sit and reach, back scratch, and single leg back bridge tests and body composition were assessed at the beginning and completion of the training period. Linear mixed models showed no significant changes in body composition, whereas flexibility (*p* < 0.001), strength (*p* < 0.001), and muscle quality index (*p* = 0.003) changed independently from the treatment. When data modification has been analyzed according to sub-sample membership, no significant differences have been observed. Age, radiation therapy, and chemotherapy seem to have independent effects on several investigated variables. Twelve weeks of adapted myofascial exercise and Nordic Walking led to significant changes in flexibility, strength, and muscle quality in breast cancer survivors, with no apparent superiority of one approach over the other.

## 1. Introduction

Surgeries, chemotherapy, radiation therapy, anti-human epidermal growth factor receptor 2 treatment, and hormonal therapy are the main traditional breast cancer management options, causing a considerable amount of morbidity in recipients. Surgeries, such as sentinel lymph node biopsy, lumpectomy, quadrantectomy, and modified radical mastectomy, with or without axillary lymph node dissection, can cause pain, postural impairments, neck mobility, upper limb anatomy, and upper body biomechanical functions. The main negative side effects are breast cancer-related lymphedema and shoulder mobility limitations, including scapula and scapulohumeral rhythm [1], which are negatively influenced by both chemotherapy [2] and radiation therapy [3].

Concerning the kinesiological negative side effects, many standard chemotherapy regimens, including platinum agents and taxanes, can induce peripheral neuropathy, resulting in a predominant sensory axonal involvement (i.e., paresthesias, pain, muscle weakness, and ototoxicity), although motor dysfunctions are also reported (i.e., *reduction or absence* of deep tendon reflexes, distal weakness and muscular atrophy, tremors, and cramps) [4,5].

Moreover, fibrosis is a common and disabling condition characterized by the abnormal formation of fibrous connective tissue leading to structural and functional changes affecting the skin, underlying fascia, muscles, organs, and bones. These result in decreased joint range of motion, mainly of the shoulder, pain, lymphatic and vascular dysfunction, as well as breast hardening, retraction, and fixation [3].

Physical exercise is widely recognized as a pillar for preventing the kinesiological negative side effects of surgeries, chemotherapy, and radiation therapy, as well as those of other pharmacological treatments [1,2,3,6,7,8]. Specifically, to counteract the kinesiological negative side effects of traditional therapies, both the literature and clinical practice underline that physical exercise should be multimodal, i.e., include aerobic, strength, flexibility, proprioceptive, and balance exercises [1,2,3,6,7,8].

Among these multimodal approaches, Nordic Walking (NW) is gaining growing attention for breast cancer survivors (BCS) [9], as it has been shown useful against several conditions. Nordic Walking has been shown to be able to decrease lymphedema [10,11,12,13,14], pain, and depression [15], and to increase upper body and trunk strength [14,16,17], shoulder range of motion [18], perception of morbidity, and disability of the shoulder [18] together with posture [16,17] and aerobic fitness [13]. The positive effects of NW on body composition [19,20], endocrine [20], biomechanical [21], cardiovascular [22], pulmonary [23], and miscellaneous [24,25] fields have been underlined in other conditions. In other words, NW seems useful to maintain/improve the kinesiological health of BCS, along with other aspects. It is interesting to note that the “NW package” for BCS, which includes a supervised and adapted introduction to NW, warm-up, NW practice, and cool-down, can actively engage the myofascial meridians by being a form of brisk walking with walking poles for further engagement of the trunk and upper limbs.

According to Myers [26], the muscles of the human body do not function as independent units. Instead, they are regarded as part of a tensegrity-like, body-wide network, with fascial structures acting as linking components, i.e., as myofascial meridians. Because fascia can transmit tension to other tissues [27,28], the adoption of this point of view is of particular importance as myofascial meridians could be responsible for disorders and pain radiating to remote anatomic structures, especially in BCS, being subjected to several local treatments (i.e., surgery and radiation therapy) which in turn affect (i.e., stiffening) the same region, spreading its response to continuous and contiguous regions. Accumulating evidence from dissection studies confirms the role played by the myofascial meridians identified by Myers [26], which connect distant parts of the body via muscles and fascial tissues [29,30]. This anatomical and functional knowledge suggests that physical exercise should be framed as a whole-body rather than a local approach to have a faster, more balanced, and effective result [31].

Summarizing, the gap in literature concerns (a) the effects of adapted myofascial exercises (ME) on the kinesiological characteristics (i.e., flexibility and strength) of BCS, based on Myers’ principles, including the dynamic engagement of the meridians, notwithstanding the well-known effects of other similar disciplines [32,33,34], and (b) the comparison with those elicited by NW practice, mainly linked with the brief lives of the cited disciplines.

Therefore, the aims of this study were to investigate, in BCS, the effects of a 12-week intervention of adapted NW or ME exercises on flexibility, strength, and body composition and to verify whether the studied disciplines elicited different results. Our starting hypotheses were: (a) adapted NW would have increased flexibility and strength, even if adapted ME would have determined better effects on them than adapted NW; (b) adapted NW would have determined better effects on body composition than adapted ME.

## 2. Materials and Methods

### 2.1. Study Design

**Figure 1:**
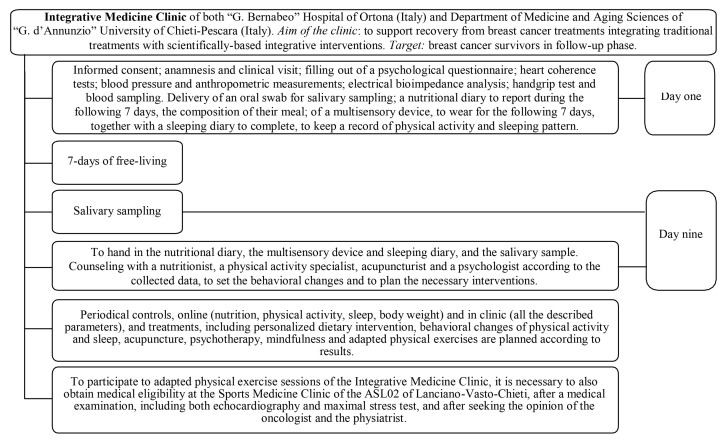
The Integrative Medicine Clinic organization.

The study was a non-randomized, parallel cohort study based on the patients of the Integrative Medicine Clinic of both the “G. Bernabeo” Hospital (Ortona, Italy) and the Department of Medicine and Aging Sciences of the “G. d’Annunzio” University of Chieti-Pescara (Italy). As displayed in Figure 1, the Integrative Medicine Clinic provides integrative support for BCS during the follow-up phase through integrative personalized interventions. The clinical mission is to direct patients regarding physical activity, sleeping, body composition and nutrition, acupuncture, analysis and control of blood and salivary metabolic, immune, and endocrine parameters, psychotherapy, mindfulness, and both adapted and supervised physical exercise both indoors and in the natural environment. After completing a multidisciplinary examination (T_0_) (Figure 1), participants willing to participate in adapted and supervised physical exercise were tested for medical eligibility by a panel of physicians (i.e., an oncologist, physiatrist, and sports medicine specialist).

The T_0_ multidisciplinary examination included a comprehensive battery of tests aimed at characterizing lifestyle (i.e., objective recording of physical activity, sleep, and sedentary behaviors plus subjective recording of nutritional habits), body composition (i.e., anthropometrics and bioelectrical impedance analysis), cardiovascular and metabolic health (i.e., blood pressure, resting heart rate, heart rate variability, and plasma metabolic analytes), adrenal balance (i.e., salivary cortisol and dehydroepiandrosterone sulfate), psychological status (i.e., anxiety, depression, psychological distress, fatigue through validated questionnaires), and quality of life (i.e., SF-36 questionnaire). After being cleared for medical eligibility to participate in the supervised and adapted workouts, physical fitness tests (i.e., balance, flexibility, strength, and aerobic capacity through field tests) were also carried out. When the T_0_ tests were executed more than 1 month prior to the beginning of the workouts, they were repeated to obtain updated basal measurements. All the tests were repeated at the end of the 12-week workout period (T_1_). To fit with the aim of the study, we will just describe the tests relevant to it, i.e., anthropometry and electrical bioimpedance analysis to characterize study participants, monitor body re-composition, and quantify muscle quality; back scratch and sit and reach to assess the effects of workouts on flexibility; handgrip and single-leg back bridge tests to assess the effects of workouts on strength. The participants considered in this study participated in the adapted and supervised workouts of the clinic concerning adapted NW or ME. Participants had an allocation based on the season and on the obtainment of the multidisciplinary eligibility for one of the disciplines proposed in the starting season (i.e., adapted walking or NW in spring and summer; adapted ME or resistance training in autumn and winter).

### 2.2. Study Participants

Inclusion criteria to participate in the intervention were: age between 30 and 70 years; 6–48 months after breast surgery; current hormone therapy; medical eligibility for adapted NW or ME practice. Exclusion criteria were: current chemotherapy; current radiotherapy; past or current diagnosis of endocrine disease; current diseases limiting NW or ME practice; and participation in any exercise program within the six months preceding the present study. The term “current” refers to the date of the basal evaluation of each participant. One hundred and sixty BCS (mean age: 52.85 ± 7.26 years) were included in the study. The study took place from January 2017 to December 2019 at the “G. Bernabeo” Hospital (Ortona, Italy) and at the “G. d’Annunzio” University of Chieti-Pescara (Chieti, Italy). The local Ethics Committee approved this study (prot. #312/2015) and participants gave their written informed consent.

### 2.3. Interventions

#### 2.3.1. Adapted NW Workouts

Ten lessons of the NW technique and 26 lessons of complete NW technique practice, with a frequency of 3 times a week (for a total of 12 weeks of training), both combined with the Isa method [10,11], were conducted and supervised by two kinesiologist instructors of the International Nordic Walking Association (INWA), specialized in physical exercise for BCS. Each lesson followed the same scheme: a 15 min warm-up, 45 min for the central phase, and a 10 min cool-down. The group performed the Isa method during the warm-up and both the Isa method and the stretching exercises during the cool-down. The Isa method [10,11] included a series of dynamic exercises, propaedeutic for NW, lymphoedema, and arthralgia, specifically designed for BCS. This required the use of Isa balls, i.e., 6- or 7-cm-diameter foam balls of different densities that can be used either alone or when applied to NW poles. The series of exercises had the following objectives: to warm up the joints gently, to reduce muscular tension, and to counteract or prevent upper limb lymphoedema. The training scheme started with exercises for hands and wrist joints that were performed using only the Isa balls. These were followed by multi-joint exercises (i.e., wrists, elbows, shoulders) that were carried out using both the NW poles and the Isa balls applied to them, and by neck exercises that were performed using only the NW poles. The main task during the workout with the Isa balls was to squeeze them gently for the whole duration of each exercise to promote the pumping effects in the upper limbs. Abdominal respiration was requested, at least, during the upper limbs and neck exercises. Afterward, trunk and lower limb exercises were carried out using the NW sticks only. Lower limb exercises were performed in a distal-to-proximal order.

The central phase of the first 10 lessons included exercises for learning the NW technique following the INWA scheme. Moving from the first to the tenth lesson, the central phase shifted its main content from the practice of single exercises to the practice of the complete NW technique. In the central phase of the 26 lessons concerning the complete NW technique, participants trained at different intensities according to time and Borg’s rating of perceived exertion scale (RPE) [35], focusing their attention on the correctness and fluidity of the complete INWA NW technique. The kinesiologists also recorded attendance at the lessons and the occurrence of injuries. During the maximal stress test, the participants were introduced to the RPE scale in general. They were also given an analytical rundown of the intensities they utilized in the 1st (i.e., 10–11 RPE), 5th (i.e., 12–13 RPE), and 8th (i.e., 13–14 RPE) week of training following Borg’s RPE scale instructions [35] to also periodically check exercise intensity. From the 1st to the 4th week, participants trained at 10–11 on the RPE scale, from the 5th to the 8th week at 12–13 on the RPE scale, and from the 9th to 12th week at 13–14 on the RPE scale. Workouts have been held in the life paths of the “G. Bernabeo” Hospital (Ortona, Italy) and of the “G. d’Annunzio” University of Chieti-Pescara (Chieti, Italy).

#### 2.3.2. Adapted ME Workouts

The adapted ME workouts followed the same general scheme, duration, frequency, and supervision as the NW workouts. Both the warm-up and central phase included global exercises, principally following the principles of Thomas W. Myers [26], while the cool-down included relaxing respiratory exercises in the supine position. The warm-up was used to gently refresh the main contents of the previous lessons, which were the base of the new lesson. The warm-up of the first lessons was composed of exercises introducing the content of the lesson. Specifically, the progression of the adapted ME is described in Table 1, and exercises were practiced in a dynamic way without interruption (except for didactic purposes) and with fluidity, control, and precision from the beginning to the end of each lesson. The exercise intensity was modulated by increasing or decreasing the entity of the active engagement in the training phases (Table 1). Workouts were conducted and supervised by two kinesiologists who specialized in physical exercise for BCS and also specialized in myofascial exercises. The kinesiologists also recorded the attendance at the lessons and the occurrence of injuries. The workouts were held in the gyms of the “G. Bernabeo” Hospital (Ortona, Italy) and the “G. d’Annunzio” University of Chieti-Pescara (Chieti, Italy).

### 2.4. Outcomes

#### 2.4.1. Anthropometrics

A third-level anthropometrist of the International Society for the Advancement of Kinanthropometry (ISAK) [36] performed body measurements on the participants while in a fasting condition. Body weight and stretched stature were measured to the nearest 0.1 kg and 0.1 cm, respectively, with the participants dressed in light clothing and without shoes, using a stadiometer with a balance-beam scale (Seca 220, Seca, Hamburg, Germany). The body mass index (BMI) has been calculated as follows: body mass (kg)/stature (m^2^).

#### 2.4.2. Electrical Bioimpedance Analysis

The body composition was assessed with an electrical bioimpedance technique using a hand-to-foot 50 kHz frequency bioelectric impedance analyzer (BIA 101, Akern, Pontassieve, Italy). The Bodygram software (Akern, Pontassieve, Italy), together with the raw data of bodily resistance and reactance, provided data concerning fat mass index (i.e., kg of fat mass/stature (m^2^)) (FMI), fat-free mass index (i.e., kg of fat-free mass/stature (m^2^)) (FFMI) [37], kg of muscle mass, muscle quality index (i.e., handgrip result test (kg)/kg of muscle mass), total body water and its balance between the intracellular and the extracellular compartment, and both phase angle and its standardized form (i.e., phase angle adjusted according to the age). The test was performed 2 h after waking and immediately after voiding, with the participants in a supine position and without conducting garments. Ten minutes of supine rest were observed before executing the test in a standardized room.

#### 2.4.3. Kinesiological Evaluations

- Back scratch test

The back scratch test [38] measures flexibility in the shoulder joints and shoulder arc both on the right and left side. Participants started the test in the standing position, placing one arm/hand on the lower back and moving it up the spine toward their head. The opposite arm/hand was placed behind their neck, moving it down the spine, aiming to place the long finger of each hand as near to the other as possible or to overlap the other hand as much as possible. The procedure was repeated reversing the arm/hand order. The gap between the fingertips of the long fingers of both hands was measured to the nearest half cm using a rigid centimeter rod. Positive numbers were used if the fingers overlapped and negative numbers if the fingers did not meet. The task was to practice two times, and then test three times for each limb in an alternate manner. The best measurement for each limb was considered.

- Sit and reach test

The sit and reach test [39] measures the flexibility of the lower back and hamstring musculature. A standardized box (i.e., the length of the top of the box was 53.3 cm and the height was 32.5 cm) was placed against a wall, and the participants sat on the floor with their knees and upper body straight and their heels against the box without shoes. The participants reached forward as far as possible along the measuring tape atop of the box, with one hand on top of the other, sliding along the box with the back and legs straight. The measured value, after being held for two seconds, was recorded with precision to the nearest mm. The point where the feet met the box was set at 0 cm from the box’s edge, and the recorded result was 0 cm plus or minus the distance from point zero. One attempt was carried out; the best result of three trials was considered. A one-minute rest interval between the measurements was applied.

- Single-leg back bridge test

The single-leg back bridge test [40] was performed with the participant lying supine with their arms across their chest, knees in flexion, and feet flat on the ground. The participant performed a double-leg hip bridge, reaching a posterior 90-degree angle of the knee joint, and once a neutral spine and pelvis position were achieved, it was requested to extend one knee (randomly determined) so the leg of the participant was straight and her thighs were parallel to one another. The participant was asked to hold this position as long as possible. The test was terminated when the participant was no longer able to maintain a neutral pelvic position, as noted by a 10-degree change in transverse or sagittal plane alignment. Pelvic positioning in the transverse plane was monitored by a digital inclinometer attached to a belt that was tightly secured to the individual’s pelvis. One attempt was carried out for each side. Two trials were performed on each side, and the average of each side was used for subsequent analyses. Three minutes of recovery were allowed between test repetitions.

- Handgrip test

The handgrip test [41,42] measures the maximum isometric strength of the hand and forearm muscles. It was performed by using an electronic hand dynamometer (EH101, Camry Electronic Zhongshan, Zhongshan, China). Each participant was seated with a straight posture and bent their elbow to 90° with the wrist maintained in a neutral position. The test was executed three times with the dominant hand and three times with the non-dominant hand, with a one-minute rest interval between the measurements. The best recorded value for each side was considered for the data analysis. During the measurement, the tester constantly encouraged participants to exert their full effort. The sum of dominant and non-dominant results determined the total handgrip result.

### 2.5. Sub-Sample Allocation

**Figure 2:**
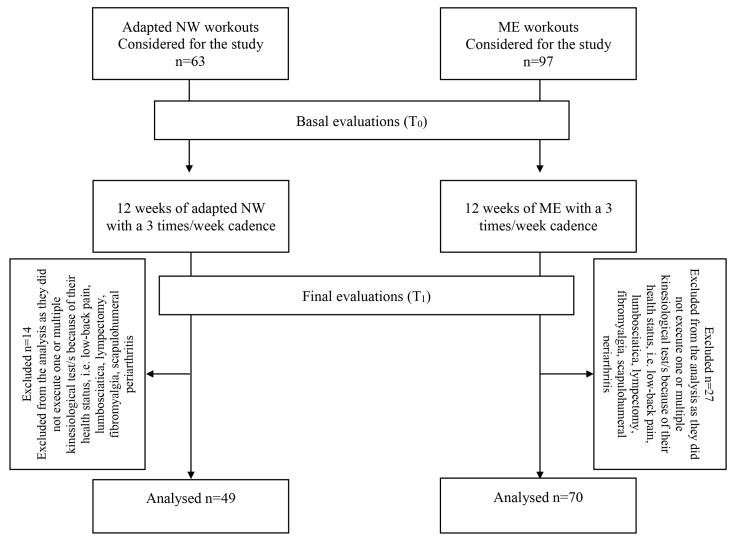
Study design.

From January 2017 to December 2019, the participants were allocated to the adapted NW sub-sample during the spring and summer seasons, when eligible for the discipline, while in the remaining seasons, they were allocated to the adapted ME, when eligible for the discipline. As shown in Figure 2, for this study, we considered 160 BCS who proved eligible to adapted NW (*n* = 63 participants) and ME (*n* = 97 participants) workouts. Fourteen members of the adapted NW workouts and 27 of the ME workouts were excluded from the analysis because their baseline health status was affected by conditions (i.e., low-back pain, lumbosciatica, lympectomy, fibromyalgia, scapulohumeral periarthritis) preventing them from executing one or more kinesiological test/s in its/their standard version, even if they were eligible to practice physical exercise.

### 2.6. Statistical Analysis

Descriptive data are shown as mean ± standard error (SE) and as absolute numbers and percentages for continuous and categorical variables, respectively. Baseline differences between the two treatment groups were assessed using an analysis of variance and chi-square test. Linear mixed models with random intercept and random slope were applied using time since baseline as the time scale. Time, group treatment, time for group treatment interaction, age, radiotherapy, and chemotherapy were considered in the model as predictors to assess differences between treatments in body composition and functional tests. SAS version 9.4 for Windows (SAS Institute, Inc., Cary, NC, USA) was used for all data processing and statistical analyses. Statistical significance was set at the conventional threshold at *p* < 0.05 (2-sided).

## 3. Results

No injuries or drop-outs were recorded. The mean workout attendance was 79.5 ± 21.9%, with no significant differences in treatment adherence between the sub-samples.

As shown in Table 2, sub-samples differed for age and radiation therapy: participants in the adapted NW sub-sample were younger and had a higher prevalence of radiation therapy than the adapted ME sub-sample.

Linear mixed models showed body composition and fitness modifications according to sub-sample membership and according to the effects of age, chemotherapy, and radiation therapy (Table 3).

When data were analyzed according to time, no significant modification of body composition was observed, while significant changes in flexibility (right and left back scratch and sit and reach tests, *p* < 0.001), strength (right and left handgrip, total handgrip, right and left single-leg back bridge, *p* < 0.001) and muscle quality index (*p* = 0.003) were observed.

When data were analyzed according to sub-sample membership, no significant differences were observed, and the same results were obtained when the data were analyzed according to the interaction of time and sub-sample membership. Age seems to negatively affect reactance (*p* = 0.004), phase angle (*p* = 0.02), back scratch—left (*p* = 0.02), and sit and reach (*p* = 0.02). Radiation therapy seems to affect just right back scratch test results (*p* = 0.04), while chemotherapy seems to affect body mass (*p* = 0.001), BMI (*p* = 0.006), body composition (i.e., standardized phase angle, *p* = 0.003; total body water, *p* = 0.001; extracellular water, *p* = 0.03; muscle mass, *p* = 0.001; FMI, *p* = 0.001 and FFMI, *p* = 0.02), muscle quality index (*p* = 0.02), and the single-leg back bridge tests results (right, *p* = 0.001; left, *p* = 0.004) (Table 3). When the linear mixed model was repeated, considering the radiation therapy as a categorizing variable, a worse back scratch—right result was shown in BCS without radiation therapy than the others, while the intervention improved the result in both sub-samples (Table 4).

Considering chemotherapy as the categorizing variable, the linear mixed model showed worse BMI, FMI, muscle quality index, and single-leg back bridge test results in BCS with chemotherapy than the others. On the contrary, BCS with chemotherapy had a higher total and extracellular body water, muscle mass, and FFMI and a better standardized phase angle than the others (Table 5).

Due to the fact that (a) total body water influences FMI, FFMI, and muscle mass estimation in the electrical bioimpedance analysis; and (b) due to their small modifications, suggesting that the observed FMI, FFMI, and muscle mass increase or decrease are not real but just an effect of total body water trend, we focused our attention on the following variables, according to the sub-sample chemotherapy membership: standardized phase angle, muscle quality index, and single-leg back bridge tests results. Notwithstanding their best starting values, BCS without chemotherapy had a greater increase in muscle quality index and single-leg back bridge test results than the others. The opposite has been shown for standardized phase angle. No significant effects have been shown for hand dominance, side and typology of surgery, hormonal therapy, and time from surgery.

## 4. Discussion

To recall of the aims of the study is the first step to better follow the contents of this section. The aims of our study were to investigate the effects of a 12-week intervention of adapted NW or ME exercises on flexibility, strength, and body composition and to verify whether the studied disciplines elicited different results. The most important finding of our study is represented by the fact that 12 weeks of supervised adapted NW, being a primarily aerobic discipline, elicits the same positive effects on whole-body flexibility (i.e., back scratch and sit and reach tests), strength (i.e., handgrip and single-leg back bridge tests), and muscle quality index as adapted ME in BCS.

In our opinion, the explanation of the same flexibility, strength, and muscle quality effects of adapted NW and ME is linked with the continuous whole-body engagement of the studied disciplines, eliciting the use of the myofascial meridians identified by Myers [26] in a different way. Indeed, during the adapted NW practice, all the meridians are contemporaneously and naturally engaged during walking, focusing attention on the proper gait cycle, on the active use of feet and posture, as well as on girdles swing and the upper limbs, while pushing on the ground through poles and properly coordinating the open–close cycle of hands [43]. On the contrary, during the adapted ME, the meridian stimulation has an analytic, progressive, and hierarchical approach in a dynamic way (Table 1) [26]. Probably, the warm-up and cool down exercises of the adapted NW also played an important role in determining the observed results, as they can stimulate the myofascial meridians with degrees of movement very similar to those reached during the adapted ME, probably closing the flexibility gap between the disciplines. In fact, in women, the average ranges of motion for the shoulders, hips, knees, and ankles during NW practice are approximately a mean value of 13° (flexion–extension), 54° (flexion–extension), 72° (flexion–extension), and 54° (dorsi–plantarflexion), respectively [44], whereas in adapted ME practice, the amplitude of the movements increases gradually until it reaches the physiological limits of the mentioned joints (but not only) through multi-joint movements involving the lines listed in Table 1. However, both adapted NW and ME included movements stimulating sensory refinement, slow-motion stretch, and elastic recoil in a fascial-oriented direction [45].

Our results provide important confirmation on the effects of age, radiation therapy, and chemotherapy on several investigated variables and reinforce the concept of the importance of physical exercise practice in BCS.

Age seems to negatively influence phase angle and both back scratch and sit and reach test results. In the literature, phase angle, a proxy index of cell membrane integrity and function, has been shown to be linked with age and has been suggested to be a prognostic, health, functional, and nutritional indicator in various diseases [46]. In patients with different types of cancer, the literature has shown that a low phase angle is associated with an impaired nutritional and functional status, decreased quality of life, and increased morbidity and mortality [46]. Moreover, back scratch and sit and reach performances have been shown to be linked with age, without physical exercise practice, due to the normal process of aging, and its management is an important aspect for the prevention and treatment of frailty [47]. Therefore, in BCS, physical exercise practice is crucial because it helps to manage some important functional variables (i.e., phase angle and flexibility) negatively affected by the natural aging process [46,47] but also by the treatments of breast cancer (i.e., surgery, chemotherapy, radiation therapy, and hormonal therapy).

Radiation therapy seems to influence the results of the back scratch—right test (i.e., of the flexibility). We cannot consider this result in an absolute way, although we could interpret it as an indicator, especially if we consider it together with the absence of significant effects of hand dominance and both sides and typology of surgery. Taken all together, these results remind us of the plasticity of our body, compensating for the same stimulus in a different kinesiological way, according to the combinations of our own characteristics and external interventions. Indeed, the literature shows several possible postural compensations according to the combination of age, typology and time of treatment, previous pathologies, and psychological impact [48,49,50].

Chemotherapy seems to be linked with basal body composition and strength. Due to the absence of data concerning the investigated variables before and after chemotherapy treatments, and the presence of integrative treatments during chemotherapy, together with their characteristics and attendance, we cannot discuss the basal data. On the contrary, we can discuss the pre–post results showing that after the proposed intervention, independent of the typology of exercise, BCS who had chemotherapy showed a greater increase in standardized phase angle, lower limb strength, and muscle quality index than BCS who did not have. Specifically, the described greater increase in BCS who had chemotherapy should be linked with their greater decrease during chemotherapy [46,51,52].

The interpretation of the absence of a significant body re-composition is linked with our previous results. Indeed, body re-composition is the result of a dynamic system composed of dietary habits, sleep, sedentary time, physical activity, and physical exercise practice. Therefore, the introduction and control of just one or two of the listed variables (i.e., physical exercise and/or dietary habits) does not provide the possibility to really know how each person consciously (or unconsciously) modifies (or not), and in which direction, all the other variables determining a different body re-composition.

Our results have intriguing practical applications, although they must be interpreted in light of the study’s limitations. The first study limitation is linked with the absence of a sub-sample random allocation. Indeed, our study participants had an allocation based on the season and on the obtaining of the multidisciplinary eligibility for one of the disciplines proposed in the starting season (i.e., adapted walking or NW in spring and summer; adapted ME or resistance training in autumn and winter). This was mainly due to the objective to reduce the dropout linked with the weather [53], especially in BCS [54], and to increase the possibility of positively affecting their health through exercise, as suggested by the current literature. The second study limitation, i.e., the final unbalance between the sub-samples, is linked with the presence of a comorbidity, not limiting the practice of adapted NW or ME, but limiting the execution of some of the chosen tests. The sample size of our study was higher than others investigating flexibility and/or strength in BCS practicing NW and reached the necessary minimum established by the existing literature [14,16,18], even if the chemotherapy sub-sample is smaller. Certainly, the non-exclusion of 41 people from the statistical analysis would have allowed us to avoid the problem of the small size of the chemotherapy sub-sample and the resulting bias. In the end, it cannot be ruled out that some of the results obtained may have been influenced differently, depending on the discipline practiced, by one or more components of the multidisciplinary approach of our clinic (Figure 1), even though the two groups had the same multidisciplinary approach and only differed in the type of physical exercise they performed.

Keeping in mind the strengths and weaknesses of our study, we can illustrate the practical applications deriving from our results. In our opinion, our results are very meaningful as they provide more choices to BCS willing to practice physical exercise by increasing the number of disciplines considered useful for their health. Our findings assist kinesiologists in tailoring the training regimen to BCS’ health status, preferences, financial situation, and availability of time, as well as taking the season into consideration. This is a very significant component as it increases the possibility of matching the therapeutic needs with the preferences of a person, producing a greater adherence to the workouts and consequently greater effectiveness of the intervention [55]. Furthermore, the concepts and training programs we have employed further support kinesiologists in their work of organizing and carrying out customized training sessions.

## 5. Conclusions

Our results add evidence to the literature concerning the significant and equivalent effects of adapted ME and NW on flexibility, strength, and muscle quality in BCS after 12 weeks of supervised training. Obviously, our results do not allow us to declare that the two disciplines are completely equivalent in determining the improvement of flexibility, strength, and muscle quality, as our intervention lasted just 12 weeks. Therefore, it is not possible to establish whether a longer period allowing the experimentation of more complex and/or intense workout progressions could determine best results with adapted ME or NW practice. The aforementioned highlights the necessity of conducting further research on the acute and long-term effects of adapted ME and NW to fully account for the effects of training progression from the initial conditioning phase to the maintenance phase. The study of the effects of a workout program including the combined use of both adapted ME and NW is another future direction. Furthermore, our findings emphasize how important is to carry out research studying and comparing the impacts of various disciplines on every aspect of BCS’ psychophysical health in order to increase the number of prescriptive alternatives for improving exercise adherence and optimizing the advantages of engaging in physical workouts. Indeed, increasing evidence underlines the importance of considering both barriers and motivations to physical exercise practice, in both indoor and outdoor disciplines, to increase adherence and to reach and optimize positive psychophysical results. Thus, we are hoping that the scientific literature will be implemented this manner as well. Our results also confirm the importance of the two disciplines against the negative effects of age, radiation therapy, and chemotherapy on the investigated variables.

## Figures and Tables

**Table 1:** Contents of the adapted ME workouts.

Weeks	Main Content of the Central Phase of Each Lesson
1	Body awareness and feet myofascial release
2	Body awareness, feet myofascial release, and their connection with pelvic floor
3	Feet myofascial release and their connection with posterior and anterior superficial lines
4	Back and front superficial lines and arm lines
5	Lateral lines and their connection with arm lines, feet myofascial release, and pelvic floor
6	Back and front superficial lines and both lateral and arm lines
7	Lateral spiral and arm lines
8	Back and front superficial lines, lateral, spiral, and arm lines
9	Feet myofascial release and their connection with pelvic floor and arm lines
10	Back and front superficial lines, lateral and arm lines
11	Back and front superficial lines, lateral, arm, and deep lines
12	Back and front functional lines and arm lines

**Table 2:** Basal characteristics of the sub-samples.

	Adapted NW (*n* = 49)	Adapted ME (*n* = 70)	*p*
Age (yrs)	49.24 ± 5.55	54.49 ± 7.43	<0.001
Right hand dominance (n)	37/49 (75.51%)	56/70 (80.00%)	0.56
Surgery side			0.11
Right	27/49 (55.10%)	25/70 (35.71%)	
Left	19/49 (38.78%)	42/70 (60.00%)	
Bilateral	3/49 (6.12%)	3/70 (4.29%)	
Types of surgery			0.74
Quadrantectomy	16/49 (32.65%)	21/70 (30.00%)	
Resection	20/49 (40.82%)	29/70 (41.43%)	
Mastectomy	13/49 (26.53%)	20/70 (28.57%)	
Lymphectomy	17/49 (34.69%)	14/70 (20.00%)	
Months from surgery	10.35 ± 4.22	11.90 ± 4.85	0.08
Chemotherapy (n)	13/49 (26.53%)	14/70 (20.00%)	0.40
Radiation therapy (n)	48/49 (97.96%)	40/70 (57.14%)	<0.001
Hormonal therapy (n)			0.016
Antiestrogens	8/49 (16.32%)	17/70 (24.28%)	
Antiestrogens + GnRH analogues	31/49 (63.26%)	17/70 (24.28%)	
Aromatase inhibitors	10/49 (20.42%)	36/70 (51.44%)	
Adherence to exercise (%)	82.64 ± 18.96	76.56 ± 25.95	0.17

Note. GnRH: Gonadotropin-releasing hormone.

**Table 3:** Linear mixed models. Body composition and fitness modification according to sub-sample membership and according to the effects of age, radiation therapy, and chemotherapy.

	Adapted NW (*n* = 49)		Adapted ME (*n* = 70)		p *	p **	p ***	*p °*	*p ^*	*p* ^$^
	T0	T1	T0	T1						
*Anthropometry*										
Stature (cm)	160.89 ± 3.65	-	159.83 ± 5.12	-	-	0.21	-	-	-	
Body mass (kg)	65.66 ± 14.04	65.35 ± 13.61	68.14 ± 13.10	67.80 ± 13.39	0.66	0.23	0.96	0.70	0.58	0.001
Body mass index (kg/m^2^)	24.80 ± 4.70	24.43 ± 4.59	27.02 ± 5.21	26.79 ± 5.37	0.42	0.04	0.73	0.93	0.45	0.006
*Body composition*										
Resistance (Ω)	574.65 ± 65.61	580.22 ± 69.25	558.53 ± 74.21	570.10 ± 78.51	0.97	0.15	0.28	0.16	0.13	0.03
Reactance (Ω)	54.57 ± 7.16	55.96 ± 7.73	51.66 ± 7.94	53.63 ± 8.47	0.58	0.19	0.49	0.004	0.29	0.23
Phase angle (°)	5.44 ± 0.53	5.52 ± 0.52	5.29 ± 0.54	5.39 ± 0.55	0.57	0.70	0.79	0.01	0.62	0.20
Standardized phase angle	−0.94 ± 0.78	−0.85 ± 0.70	−0.85 ± 0.75	−0.77 ± 0.77	0.44	0.72	0.90	0.07	0.37	0.003
Total body water (L)	33.00 ± 3.56	32.82 ± 3.69	33.41 ± 3.76	33.01 ± 3.83	0.92	0.34	0.26	0.66	0.38	0.001
Extracellular water (L)	16.02 ± 1.95	15.80 ± 1.97	16.49 ± 2.07	16.12 ± 2.01	0.75	0.30	0.34	0.12	0.55	0.03
Muscle mass (kg)	28.31 ± 3.31	28.41 ± 3.38	28.17 ± 3.49	28.19 ± 3.66	0.63	0.59	0.72	0.44	0.33	0.001
FMI (kg/m^2^)	7.93 ± 3.61	7.90 ± 3.48	9.01 ± 3.46	9.02 ± 3.58	0.75	0.10	0.75	0.63	0.62	0.001
FFMI (kg/m^2^)	17.36 ± 1.68	17.28 ± 1.72	17.94 ± 2.01	17.78 ± 2.01	0.95	0.06	0.36	0.64	0.15	0.02
*Flexibility*										
Back scratch—right (cm)	−4.49 ± 7.22	−0.90 ± 5.95	−4.74 ± 8.28	−1.51 ± 7.03	<0.001	0.18	0.48	0.11	0.04	0.50
Back scratch—left (cm)	−8.71 ± 7.09	−3.53 ± 6.22	−8.51 ± 9.15	−3.96 ± 7.42	<0.001	0.09	0.27	0.02	0.18	0.40
Sit and reach (cm)	−0.16 ± 6.80	5.16 ± 6.68	−2.04 ± 8.46	2.91 ± 7.69	<0.001	0.73	0.63	0.02	0.14	0.29
*Strength*										
Handgrip—right (kg)	23.67 ± 4.11	26.39 ± 3.80	22.51 ± 3.92	25.30 ± 3.09	<0.001	0.25	0.88	0.99	0.91	0.39
Handgrip—left (kg)	22.61 ± 3.77	26.27 ± 3.29	21.41 ± 3.48	25.13 ± 2.72	<0.001	0.18	0.87	0.83	0.72	0.29
Total handgrip (kg)	46.29 ± 7.58	52.65 ± 6.97	43.93 ± 7.08	50.43 ± 5.65	<0.001	0.19	0.86	0.91	0.81	0.32
Single-leg back bridge—right (s)	36.71 ± 21.76	81.76 ± 33.50	29.03 ± 21.92	65.97 ± 29.48	<0.001	0.83	0.08	0.96	0.49	0.001
Single-leg back bridge—left (s)	38.00 ± 25.62	82.53 ± 32.59	33.27 ± 27.48	67.83 ± 29.05	<0.001	0.52	0.06	0.65	0.52	0.004
Muscle quality index	1.21 ± 0.20	1.38 ± 0.19	1.17 ± 0.23	1.36 ± 0.23	0.003	0.32	0.54	0.72	0.96	0.02

Note. FMI: fat mass index; FFMI: fat-free mass index. *p* *: time comparison; *p* **: sub-samples comparison; *p* ***: interaction of time and sub-sample membership; *p* °: age effect; *p* ^: radiation therapy effect, *p* $: chemotherapy effect.

**Table 4:** Linear Mixed Models. Body composition and fitness modification according to sub-sample membership. The model considers: time for group interaction, age, radiation therapy, and chemotherapy.

	No Radiation Therapy (*n* = 31)		Radiation Therapy (*n* = 88)		β ± SE *
	T0	T1	T0	T1	
*Anthropometry*					
Stature (cm)	160.89 ± 4.87	-	160.04 ± 4.49	-	-
Body mass (kg)	67.10 ± 11.09	66.50 ± 11.27	67.12 ± 14.30	66.90 ± 14.24	−1.66 ± 2.97
Body mass index (kg/m^2^)	26.55 ± 4.72	26.08 ± 4.74	25.95 ± 5.25	25.73 ± 5.34	−0.85 ± 1.12
*Body composition*					
Resistance (Ω)	574.65 ± 71.90	581.35 ± 70.57	561.83 ± 70.73	571.77 ± 76.33	24.73 ± 16.12
Reactance (Ω)	52.26 ± 7.39	54.71 ± 7.73	53.07 ± 7.88	54.55 ± 8.43	1.79 ± 1.70
Phase angle (°)	5.20 ± 0.45	5.39 ± 0.53	5.41 ± 0.56	5.46 ± 0.54	−0.06 ± 0.11
Standardized phase angle	−0.98 ± 0.66	−0.77 ± 0.73	−0.85 ± 0.79	−0.81 ± 0.75	−0.15 ± 0.16
Total body water (L)	32.92 ± 3.22	32.63 ± 3.14	33.35 ± 3.82	33.03 ± 3.96	−0.74 ± 0.82
Extracellular water (L)	16.40 ± 1.94	15.94 ± 1.91	16.26 ± 2.06	16.00 ± 2.03	−0.26 ± 0.45
Muscle mass (kg)	27.54 ± 2.68	27.86 ± 2.83	28.47 ± 3.61	28.43 ± 3.75	−0.75 ± 0.76
FMI (kg/m^2^)	8.78 ± 3.03	8.65 ± 3.15	8.49 ± 3.72	8.53 ± 3.72	−0.39 ± 0.78
FFMI (kg/m^2^)	17.54 ± 1.83	17.43 ± 1.82	17.75 ± 1.93	17.63 ± 1.94	−0.63 ± 0.42
*Flexibility*					
Back scratch—right (cm)	−7.16 ± 9.84	−3.32 ± 8.15	−3.75 ± 6.83	−0.53 ± 5.83	−3.39 ± 1.61
Back scratch—left (cm)	−10.42 ± 10.53	−5.26 ± 8.11	−7.95 ± 7.37	−3.26 ± 6.43	−2.34 ± 1.70
Sit and reach (cm)	−4.00 ± 8.77	1.71 ± 8.34	−0.31 ± 7.30	4.59 ± 6.86	−2.45 ± 1.64
*Strength*					
Handgrip—right (kg)	22.26 ± 4.73	25.52 ± 3.39	23.25 ± 3.74	25.83 ± 3.45	−0.09 ± 0.82
Handgrip—left (kg)	21.23 ± 3.80	25.10 ± 2.86	22.15 ± 3.57	25.77 ± 3.05	−0.26 ± 0.73
Total handgrip (kg)	43.48 ± 8.30	50.61 ± 6.11	45.40 ± 6.97	51.60 ± 6.38	−0.35 ± 1.50
Single-leg back bridge—right (s)	24.55 ± 14.50	66.74 ± 30.23	34.89 ± 23.68	74.49 ± 32.56	−3.98 ± 5.36
Single-leg back bridge—left (s)	27.26 ± 20.91	70.39 ± 30.22	38.02 ± 28.06	75.11 ± 31.72	−3.89 ± 5.72
Muscle quality index	1.16 ± 0.26	1.37 ± 0.27	1.19 ± 0.20	1.36 ± 0.20	0.01 ± 0.05

Note. FMI: fat mass index; FFMI: fat-free mass index. * Estimates for the differences between times. The radiation therapy sub-sample was considered as the reference group. Therefore, β ± SE assesses the mean differences between times in participants who did not have radiation therapy compared to those who had it.

**Table 5:** Linear Mixed Models. The effects of chemotherapy on body composition and fitness, according to the times of the study. The model considers: time for group interaction, age, radiation therapy, and chemotherapy.

	No Chemotherapy (*n* = 92)		Chemotherapy (*n* = 27)		β ± SE *
	T0	T1	T0	T1	
*Anthropometry*					
Stature (cm)	160.21 ± 4.43		160.44 ± 5.18	-	-
Body mass (kg)	64.83 ± 11.58	64.48 ± 11.39	74.91 ± 16.59	74.65 ± 16.96	−10.36 ± 2.79
Body mass index (kg/m^2^)	25.54 ± 4.61	25.16 ± 4.55	28.03 ± 6.25	28.08 ± 6.51	−2.93 ± 1.04
*Body composition*					
Resistance (Ω)	569.61 ± 70.12	583.30 ± 73.23	550.04 ± 73.03	543.48 ± 72.68	32.23 ± 15.02
Reactance (Ω)	53.09 ± 8.11	54.98 ± 8.47	52.07 ± 6.35	53.26 ± 7.29	1.90 ± 1.58
Phase angle (°)	5.33 ± 0.55	5.39 ± 0.53	5.44 ± 0.51	5.62 ± 0.53	−0.14 ± 0.11
Standardized phase angle	−0.97 ± 0.70	−0.91 ± 0.66	−0.61 ± 0.89	−0.42 ± 0.88	−0.46 ± 0.15
Total body water (L)	32.75 ± 3.51	32.30 ± 3.50	34.89 ± 3.78	35.08 ± 3.86	−2.53 ± 0.77
Extracellular water (L)	16.11 ± 2.04	15.78 ± 1.99	16.92 ± 1.87	16.69 ± 1.87	−0.94 ± 0.42
Muscle mass (kg)	27.73 ± 3.16	27.60 ± 3.15	29.91 ± 3.72	30.58 ± 3.86	−2.4 ± 0.70
FMI (kg/m^2^)	7.98 ± 2.99	8.04 ± 2.99	10.54 ± 4.52	10.33 ± 4.73	−2.52 ± 0.73
FFMI (kg/m^2^)	17.53 ± 1.77	17.33 ± 1.76	18.29 ± 2.22	18.39 ± 2.18	−0.98 ± 0.40
*Flexibility*					
Back scratch—right (cm)	−4.43 ± 8.09	−1.10 ± 6.84	−5.33 ± 6.93	−1.81 ± 5.73	1.05 ± 1.50
Back scratch—left (cm)	−8.27 ± 8.71	−3.64 ± 7.25	−9.70 ± 6.93	−4.26 ± 5.79	1.32 ± 1.58
Sit and reach (cm)	−0.95 ± 7.69	4.05 ± 7.32	−2.37 ± 8.39	3.11 ± 7.55	1.64 ± 1.53
*Strength*					
Handgrip—right (kg)	22.86 ± 3.75	25.53 ± 3.25	23.44 ± 4.90	26.48 ± 3.94	−0.67 ± 0.76
Handgrip—left (kg)	21.71 ± 3.20	25.42 ± 2.71	22.59 ± 4.85	26.19 ± 3.85	−0.73 ± 0.68
Total handgrip (kg)	44.57 ± 6.63	50.96 ± 5.82	46.04 ± 9.47	52.67 ± 7.70	−1.40 ± 1.40
Single-leg back bridge—right (s)	34.39 ± 23.23	77.22 ± 31.74	24.70 ± 15.80	56.30 ± 27.86	16.56 ± 4.99
Single-leg back bridge—left (s)	38.00 ± 28.45	77.76 ± 31.26	25.74 ± 16.94	60.67 ± 28.04	15.60 ± 5.33
Muscle quality index	1.20 ± 0.22	1.39 ± 0.22	1.12 ± 0.22	1.28 ± 0.19	0.10 ± 0.04

Note. FMI: fat mass index; FFMI: fat-free mass index. * Estimates for the differences between times. The chemotherapy sub-sample was considered as the reference group. Therefore, β ± SE assesses the mean differences between times in participants who did not have chemotherapy compared to those who had it.

## Data Availability

Data from the present study are available from the corresponding author upon reasonable request.
